# Correlation between immune-related adverse events and response to pembrolizumab in advanced melanoma patients

**DOI:** 10.1186/2051-1426-3-S2-P186

**Published:** 2015-11-04

**Authors:** Antonio Maria Grimaldi, Ester Simeone, Lucia Festino, Diana Giannarelli, Marco Palla, Corrado Caracò, Marcello Curvietto, Assunta Esposito, Maria Chiara Grimaldi, Nicola Mozzillo, Paolo A Ascierto

**Affiliations:** 1 O.U. Melanoma, Cancer Immunotherapy and Innovative Therapies Istituto Nazionale Tumori di Napoli Fondazione “G. Pascale”, Napoli, Italy; 2 O.U. Melanoma, Cancer Immunotherapy and Innovative Therapies Istituto Nazionale Tumori di Napoli Fondazione, Napoli, Italy; 3 Regina Elena National Cancer Institute, Rome, Roma, Italy; 4 O.U. Melanoma and Sarcoma Surgery Istituto Nazionale Tumori di Napoli Fondazione “G. Pascale”, Napoli, Italy; 5 Catholic University of Sacred Heart, Roma, Italy; 6 Istituto Nazionale Tumori Fondazione G. Pascale, Naples, Italy

## Background

Immunomodulation with pembrolizumab (anti-PD-1) has been shown to reach significant objective response (RR) and extend overall survival (OS) both in ipilimumab pre-treated and naive patients with metastatic melanoma. While this immunotherapy gives OS and OR benefits, it can also result in immune-related adverse events (irAEs) which are generally of low grade and easily manageable. We retrospectively evaluated if there was a correlation between occurrence of irAEs with OR and disease control rate (DCR).

## Methods

Inside the expanded access program, pembrolizumab was given in patients progressing after ipilimumab at dosage of 2 mg/kg every 3 weeks until PD or unacceptable toxicity. At our Institution 47 patients (25M, 22F) were treated. The median age was 49 years (range 28-70). All patients were stage M1c. The median duration of treatment was of 4.5 months (range 1-8).

## Results

At a median follow up of 3 months (range 1 – 8+), 11 (23.4%) pts had OR and 21 (44.7%) pts achieved DCR. In pts with grade 0/1 irAEs OR was 19.2% while in pts with grade ≥ 2 irAEs was 28.6%. OR was slightly higher among pts who experienced irAEs but the difference was not statistically significant (p = 0.45). Also DCR was slightly higher but not significant among those patients who experienced an irAEs (11/21, 52.4%) compared with those who did not (10/26, 38.5%) (p = 0.34). Among patients with grade 0/1 irAEs was observed 1 (3.8%) complete response (CR), 4 (15.4%) partial responses (PR), 5 (19.3%) stable disease (SD) and 16 (61.5%) progression of disease (PD), while in the group with irAEs was observed 1 (4.8%) CR, 5 (23.8%) PR, 5 (23.8%) SD and 10 (47.6%) PD.

## Conclusions

OR and DCR with pembrolizumab are similarly observed among pts who develop irAEs or not. Thus, pts who do not experience an irAE have the same probability to reach clinical benefit with pembrolizumab than those who experienced irAEs

